# Neuroprotective Effects of D-(-)-Quinic Acid on Aluminum Chloride-Induced Dementia in Rats

**DOI:** 10.1155/2020/5602597

**Published:** 2020-05-12

**Authors:** Lu Liu, Yonggang Liu, Jing Zhao, Xiaoming Xing, Chao Zhang, Huihong Meng

**Affiliations:** ^1^Department of Clinical Psychology, Baoding No. 1 Central Hospital, Baoding, Hebei 071000, China; ^2^Department of Neurology, Baoding No. 1 Central Hospital, Baoding, Hebei 071000, China

## Abstract

**Objective:**

The present study was designed to evaluate the neuroprotective effects of D-(-)-quinic acid on aluminum chloride- (AlCl_3_-) induced neurobehavioral and biochemical changes in rats. This study showed the behavioral and biochemical effects of D-(-)-quinic acid on rats with particular emphasis on the hippocampus and frontal cortex which are associated with memory.

**Materials and Methods:**

Chronic administration of aluminum chloride at a dose of 175 mg/kg, p.o. for a period of 25 days markedly increased the level of acetylcholinesterase (AChE) activity and reduced the levels of antioxidant enzymes in the brain. Two doses of D-(-)-quinic acid (200 mg/kg and 400 mg/kg) were selected based on previous safety/toxicity studies and administered orally from the 26th day to the 36th day of the trial. Behavioral parameters were assessed using the Morris water maze test and an actophotometer in rats. Biochemical parameter content and histology of brain tissue were assessed on the final day of the experiment.

**Results:**

D-(-)-Quinic acid (200 mg/kg and 400 mg/kg) orally administered alongside AlCl_3_ rescued AChE activity and the behavioral impairments caused by aluminum. There was significant inhibition of MAO-B in D-(-)-quinic acid-treated rats. Histopathological studies in the hippocampus and cortex of the rat brain also supported that D-(-)-quinic acid markedly reduced the toxicity of AlCl_3_ and preserved the normal histoarchitecture pattern of the hippocampus and cortex. These results indicate that D-(-)-quinic acid can reverse memory loss caused by aluminum intoxication by attenuating AChE activity and rescuing the deleterious effect of AlCl_3_.

## 1. Introduction

Neurodegenerative diseases such as Alzheimer's disease (AD), Creutzfeldt–Jakob disease, and familial prion disease are the most frequent causes of dementia [1, 2]. Dementia is either chronic or progressive in nature and leads to the disturbance of multiple higher cortical functions including memory, thinking, orientation, comprehension, calculation, learning capability, language, and judgment [1]. Cognitive function impairments are manifested in patients through deterioration in emotional control, social behavior, or motivational imbalance (WHO, 1992). The incidence rate for dementia increases exponentially with age, as seen in patients with AD [3].

**Figure 1:**
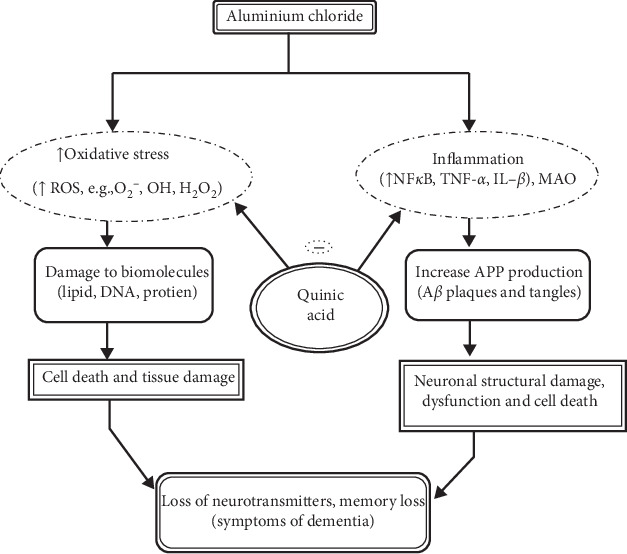
Systematic representation of the drug hypothesis for aluminum chloride-induced dementia.

Dementia is associated with reduced levels of neurotransmitters like acetylcholine. Therefore, drugs such as acetylcholinesterase inhibitors, which increase the concentration of acetylcholine in central synapses, are the most suitable choice for treatment. Three AChEIs, donepezil, rivastigmine, and galantamine, have been used to treat mild to moderately severe AD and other types of dementia [4]. Several other classes of drugs have also been studied for the prevention of memory loss and progression of neuronal damage, such as estrogen replacement therapy [5]. D-(-)-Quinic acid belongs to the class of phenolic acids which exist in a variety of plants and microorganisms [6]. They cannot be synthesized by mammals, including humans. D-(-)-Quinic acid supplementation through diet aids in the synthesis of tryptophan and nicotinamide in the gastrointestinal tract (GIT), which leads to an enhancement of DNA repair and nuclear factor kappa B inhibition [7]. D-(-)-Quinic acid has been reported to have antioxidant and anti-inflammatory properties [8]. The potential role of D-(-)-quinic acid in dementia has not been evaluated previously but is an interesting candidate as the accumulation of free radicals causes degenerative events associated with aging such as dementia. Free radicals play a major role in the pathogenesis of dementia, and antioxidants play an important role in the treatment of dementia (Figure 1). D-(-)-Quinic acid has been reported as a potential NF*κ*B inhibitor [7]. Overproduction of inflammatory mediators by the microglia can induce pathogenesis in several neurodegenerative diseases such as Alzheimer's disease, Parkinson's disease, and multiple sclerosis [8].

Here, we hypothesized that D-(-)-quinic acid would prevent the oxidation of free radicals and inhibit the release of inflammatory mediators such as NF*κ*B, TNF*α*, and NO, which are involved in inflammation and cellular stress. We also hypothesized it would help in the inhibition of the monoamine oxidase enzyme (MAO), which is a recently described therapeutic target for dementia.

## 2. Materials and Methods

### 2.1. Drugs and Chemicals

D-(-)-Quinic acid was purchased from Sigma Aldrich. Rivastigmine was a gift from a pharmaceutical company. Aluminum chloride, toluene, DTNB, acetylthiocholine iodide, trichloroacetic acid, thiobarbituric acid, and sodium carboxymethyl cellulose were purchased from Himedia Pvt. Ltd. (Mumbai) and S D Fine Chemicals Ltd (Mumbai). Methanol, chloroform, dichloromethane, tween 80, *n*-butanol, and ethyl acetate were of analytical grade (AR).

### 2.2. Experimental Animals

Sprague Dawley (180–200 gm) rats of either sex were used in this experimental work. The animals were purchased from the Vital River Laboratory Animal Technology. The animals were housed in groups of six and kept in polypropylene cages kept under controlled room temperature (22–24°C) under a 12 h light-dark cycle. The animals were kept in paddy husks. The animals were allowed free access to food and water. All procedures were approved and carried out according to the guidelines of the committee for the purpose of control and supervision of experiments on animals.

### 2.3. Experimental Design

On day 5, the rats were randomized based on ELT (escape latency) and divided into 5 groups (*n* = 5). Aluminum chloride, quinic acid, and rivastigmine solutions were made fresh each day. Rats were given aluminum chloride (175 mg/kg) orally from day 6 (i.e., 24 h after the completion of the retention trial on day 5) to day 25. This dosing regimen of aluminum chloride was selected based on previous reports because of its high rate of induction and low mortality [9]. Aluminum chloride was dissolved in distilled water and administered orally at a dose of 0.5 ml/100g body weight. The standard drug (rivastigmine 2.5 mg/kg) was suspended in 0.5% sodium carboxymethyl cellulose (CMC) and was given orally from day 26 to day 36, and the test drug quinic acid (200 and 400 mg/kg) was dissolved in distilled water and was given orally from day 26 to day 36. The control group received normal saline.

The groups were as follows:

- Group I (normal control): 0.9% NaCl 5 ml/kg, p.o., from the 6th day to the 36th day.
- Group II (aluminum chloride-induced dementia): AlCl_3_ 175 mg/kg, p.o., from day 6 to 25 + 0.9% NaCl 5 ml/kg, p.o., from the 26th day to the 36th day.
- Group III (standard drug treated): AlCl_3_ 175 mg/kg, p.o., from day 6 to 25 + rivastigmine 2.5 mg/kg, p.o., from day 26 to day 36.
- Group IV (test drug treated): AlCl_3_ 175 mg/kg, p.o., from day 6 to 25 + D-(-)-quinic acid 200 mg/kg, p.o., from day 26 to day 36.
- Group V (test drug treated): AlCl_3_ 175 mg/kg, p.o., from day 6 to day 25 + (D-(-)-quinic acid 400 mg/kg, p.o., from day 26 to day 36.

The water maze consists of a circular tank (150 cm diameter and 40 cm height). The water pool was divided into equally spaced quadrants (north-east, south-east, south-west, and north-west) along the circumference of the pool. An escape platform (10 cm diameter) submerged 2 cm below the water surface was placed in the NW quadrant. All rats were subjected to one session of four trials per day for four consecutive days (0−4th day). During each trial, the animals were placed in each quadrant to eliminate quadrant effects. All rats were left on the platform for 30 s and then removed and well dried. Rats failing to find the platform within 60 s were guided to the platform. On day 5 (probe day), 24 h after the training, the escape platform was removed and the probe trial was conducted. The cutoff time for animals to swim was set to 60 s before the end of session. Similarly, the retention trials were conducted on day 26 and day 36 to evaluate memory. Time to reach the hidden platform (escape latency) and percentage of time spent in the target quadrant (NW) was measured during the retention trials [10].

Locomotor activity was assessed in animals using a digital actophotometer. Their ambulatory movements were recorded for a period of 10 min and expressed in terms of total photo beam counts for 10 min per animal. Locomotor activity was assessed on days 5, 16, and 36 before the probe trial in the Morris water maze.

### 2.4. Evaluation

The animals were sacrificed by cervical dislocation. The whole brain was carefully removed from the skull, immediately after dislocation. For preparation of the homogenate, the fresh whole brain was weighed and transferred to a glass homogenizer and a 10% (w/v) tissue homogenate was prepared in 0.1 M phosphate buffer (pH 7.4, stored at −2–8°C). The homogenate was centrifuged at 3000 rpm for 10 min, and the resultant cloudy supernatant liquid was used for biochemical assessments.

Choline esterase enzyme determination was measured by the Ellman method [11]. 0.4 ml of the supernatant from the brain homogenate, 2.6 ml of 0.05 M phosphate buffer, and 0.1 ml of 0.1 M DTNB (5,5-dithiobis-(2-nitrobenzoic acid)) were processed, and the absorbance was measured at 412 nm through UV spectrophotometry, until the reading was constant and then 0.02 ml acetylthiocholine iodide (substrate) was added. Immediately the absorbance at 412 nm was measured for 7 minutes with one minute intervals. The results were expressed as nM/L/min/g of tissue.

### 2.5. Glutathione Estimation

GSH was measured using the method described by Ellman et al. [11–14]. The supernatant from the brain homogenate and 10% TCA (1 : 1 ratio) were added and centrifuged at 1000 rpm for 10 min. 0.05 ml supernatant, 2 ml of 0.3 M dihydrogen phosphate, and 0.25 ml of freshly prepared DTNB were added, and the absorbance was measured at 412 nm. The results were calculated as nM/mg of protein.

#### 2.5.1. Catalase Estimation

Catalase activity was measured using the Luck method [15], wherein the breakdown of H_2_O_2_ was measured. To 0.05 ml of the supernatant from the brain homogenate, 3 ml of H_2_O_2_ phosphate buffer was added and the change in absorbance was recorded for 2 min at 30 s intervals at 240 nm. The results were calculated as micromoles of H_2_O_2_ decomposed per min/mg of protein.

#### 2.5.2. SOD Enzyme Activity

SOD activity was measured using the method described by Armstrong et al. [16]. The assay system consisted of EDTA 0.1 Mm, sodium carbonate 50 mM, and 96 mM nitro blue tetrazolium (NBT). In the cuvette, 2 ml of the above mixture, 0.05 ml of supernatant from the brain homogenate, and 0.05 ml of hydroxylamine were added, and the autooxidation of hydroxylamine was measured for 2 min at 30 s intervals by measuring the absorbance at 560 nm. The results were expressed as units/mg protein.

#### 2.5.3. LPO Level Determination

In this method, 1 ml of the supernatant was taken from the centrifuged homogenate (10%) and 0.5 ml of 30% TCA was added, followed by 0.5 ml of 0.8% TBA reagent. The tubes were then covered with aluminum foil and kept in a shaking water bath for 30 min at 80°C. After 30 min, tubes were taken out and kept in ice-cold water for 30 min. These were then centrifuged again at 3000 rpm for 15 min. The absorbance of the supernatant was read at 540 nm at room temperature against a blank [17, 18].

#### 2.5.4. Nitrite Content Estimation

Nitrites were measured by using the Najmun method [19]. 0.2 ml of the supernatant from the brain homogenate was mixed with the freshly prepared Griess reagent solution, and the absorbance was measured at 546 nm. The results were calculated as nM/mg of protein.

#### 2.5.5. Monoamine Oxidase Activity (MAO)

MAO activity was assessed by spectrophotometry and followed by the previously reported method [20]. The assay mixture contained 4 mM of serotonin as a specific substrate for MAO-A, 250 *μ*l solution of the brain homogenate, and 100 mm sodium phosphate buffer (pH 7.4) up to a final volume of 1 ml. The reaction was allowed to proceed at 37°C for 20 minutes and stopped by adding 1 M HCl (200 *μ*l). The reaction product was extracted with 5 ml of butyl acetate, and the organic phase was measured at a wavelength of 280 nm using a spectrometer. Blank samples were prepared by adding 1 M HCl (200 *μ*l) prior to the reaction before the same process was carried out.

### 2.6. Statistical Analysis

The data were analyzed using Graph Pad Prism 7.0 software, and the data obtained were expressed as mean ± SEM. followed by a two-way analysis of variance (ANOVA) or one-way ANOVA when applicable; individual comparison was done using Tukey's multiple comparison test for statistical significance set to *p* < 0.001.

#### 2.6.1. Histopathology Study of the Rat Brain

The cortex and hippocampus of the control and experimental rats were fixed in 4% formalin and embedded in paraffin. Next, they were sliced into 5 *μ*m sections using a section cutter (Leica, Germany). The sections were stained with hematoxylin and eosin (H&E) and examined under a light microscope.

**Figure 2:**
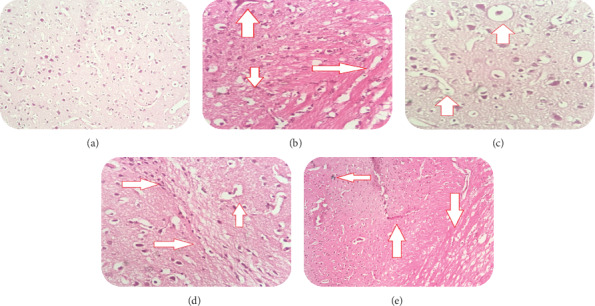
Histopathological changes in the rat brain which showed neuronal degeneration. (a) Group I. (b) Group II. (c) Group III. (d) Group IV. (e) Group V.

*(1). Observations*. From the histopathological study, it was observed that the normal control group (Group I) showed a normal brain parenchyma with normal neuronal morphology. Aluminum chloride animals (Group II) showed neuronal degradation in the brain parenchyma, small pyknotic nuclei, and extracellular eosinophilic deposition. In the standard group, animals (Group III) showed mild changes in neuronal degeneration, and many nuclei were pyknotic and closely packed. Group IV (D-(-)-quinic acid 200 mg/kg) showed focal areas of cellular degeneration. However, some areas were morphologically normal. Group V (D-(-)-quinic acid 400 mg/kg) showed improvement in the near-normal brain parenchyma with minimal degenerative changes (Figure 2).

## 3. Results

### 3.1. Effect of D-(-)-Quinic Acid on Aluminum Chloride-Induced Behavioral Parameters

#### 3.1.1. Effect of D-(-)-Quinic Acid on Aluminum Chloride-Induced Dementia in Rats Using the Morris Water Maze

The rats treated with aluminum chloride showed severe toxicity in the brain, leading to spatial memory impairment, as observed in the Morris water maze test. Initially, rats spent more time in the target quadrant (NW) compared to the other quadrants during the retention trial conducted on the 5th day (probe day). The control group animals showed normal retrieval of memory until the end of the experiment (on the 36th day). Untreated aluminum chloride animals (Group II) showed significant changes in ELT (*p* < 0.001) and TT (*p* < 0.001) during the retention trial as compared to the control group. Rivastigmine-treated rats showed significant improvement in ELT and increases in TT from the 26th day to the 36th day of the trial when compared to Group II. The D-(-)-quinic acid-treated group showed a dose-dependent improvement in spatial memory from the 26th day to the 36th day of the trial when compared to the control group (Tables 1 and 2).

#### 3.1.2. Effect of D-(-)-Quinic Acid on Aluminum Chloride-Induced Dementia in Rats Using Actophotometer

The ambulatory movements of the animals were measured using a digital actophotometer. The control group showed no change in locomotion during the 5th, 16th, 26th, and 36th day of the experiment. Untreated aluminum chloride rats (Group II) showed a gradual decrease in locomotion as the experiment progressed and had a significant decrease on the 36th day when compared with the control group. The animals treated with the standard drug rivastigmine showed a significant improvement in locomotion after the 26th day of treatment compared to the negative control, Group II. D-(-)-Quinic acid when given at a dose of 200 mg/kg showed a slight increase in locomotion but no significant differences were found compared with Group II, but at a dose of 400 mg/kg, animals showed a significant increase in locomotor activity as compared to Group II (Table 3).

### 3.2. Effect of D-(-)-Quinic Acid on Biochemical Parameters

#### 3.2.1. Effect of D-(-)-Quinic Acid on AChE Level in the Rat Brain

**Figure 3:**
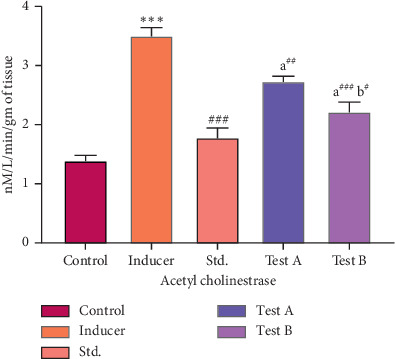
Effect of D-(-)-quinic acid on AChE levels in the rat brain. All values are expressed as mean ± SEM.

The AChE levels of the different groups are shown in Figure 3. The AChE level in the brain was found to be 1.39 ± 0.09 nM/L/min/gm of tissue. Aluminum chloride-treated rats (Group II) showed a higher level of AChE in the brains as compared to control rats. The rats treated with the standard drug (Group III) showed a significant decrease in AChE in the brain when compared to the rats treated with aluminum chloride (Group II). D-(-)-Quinic acid also showed a significant decrease in AChE level compared with Group II, which occurred in a dose-dependent manner (Table 4).

#### 3.2.2. Effect of D-(-)-Quinic Acid on GSH Levels in the Rat Brain

**Figure 4:**
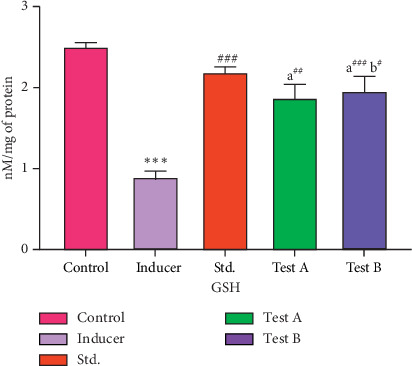
Effect of D-(-)-quinic acid on GSH levels in the rat brain. All values are expressed as mean ± SEM.

GSH levels in the normal control group (Group I) were found to be 2.50 ± 0.10 nM/mg of protein. Untreated aluminum chloride rats showed significant depletion (*p* < 0.001) in GSH levels when compared with the normal control group. Rats treated with rivastigmine showed significantly increased (*p* < 0.001) GSH levels in the rat brain tissue compared to those in Group II. Treatment with D-(-)-quinic acid (Groups IV and V) resulted in a significant increase (*p* < 0.001) in GSH levels when compared to aluminum chloride-treated animals (Group II), which once more occurred in a dose-dependent manner (Figure 4 and Table 4).

#### 3.2.3. Effect of D-(-)-Quinic Acid on Catalase Levels in the Rat Brain

**Figure 5:**
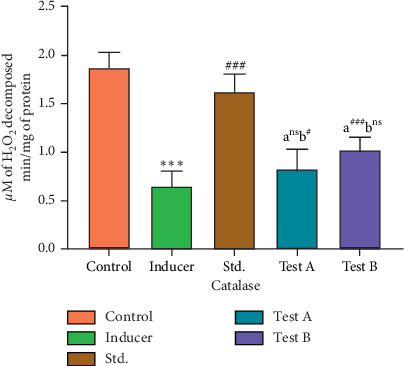
Effect of D-(-)-quinic acid on catalase levels in the rat brain. All values are expressed as mean ± SEM.

Catalase levels in control rats (Group I) were found to be 1.87 ± 0.156 H_2_O_2_*µ*M of H_2_O_2_ decomposed per min/mg of protein. Aluminum chloride-treated rats (Group II) showed a significant decrease (*p* < 0.001) in catalase levels in the brain as compared to the control group. Rats treated with the standard drug rivastigmine (Group III) showed a significant increase in catalase levels in the brain when compared to Group II. Treatment of the rats with D-(-)-quinic acid (Groups IV and V) resulted in a significant (<0.001) increase in catalase levels in the brain when compared to aluminum chloride-treated animals (Group II). D-(-)-Quinic acid resulted in higher increase at a dose of 400 mg/kg after treatment for 36 days (Figure 5 and Table 4).

#### 3.2.4. Effect of D-(-)-Quinic Acid on SOD Levels in the Rat Brain

**Figure 6:**
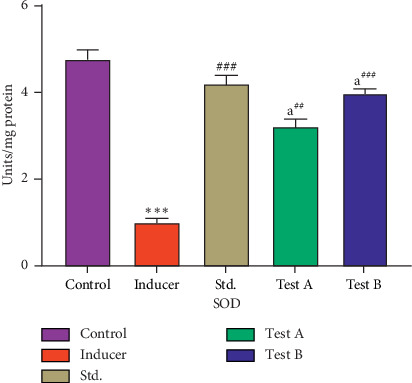
Effect of D-(-)-quinic acid on SOD levels in the rat brain. All values are represented as mean ± SEM.

Animals treated with aluminum chloride (Group II) showed significantly decreased (*p* < 0.001) SOD levels in the brain on the 36th day compared with the control group. Standard drug-treated rats (Group III) had increased SOD levels in the brain when compared with aluminum chloride-treated rats (Group II). After treatment with D-(-)-quinic acid (Groups IV and V), there was a significant (*p* < 0.001) increase in SOD levels in the brain when compared to aluminum chloride-treated animals (Group II) (Figure 6 and Table 4).

#### 3.2.5. Effect of D-(-)-Quinic Acid on TBARS Levels in the Rat Brain

**Figure 7:**
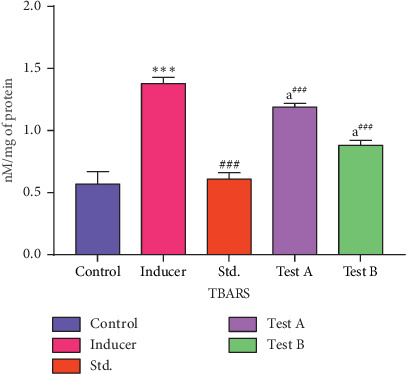
Effect of D-(-)-quinic acid on TBARS levels in the rat brain. All values are represented as mean ± SEM.

The level of TBARS in the control group (Group I) was 0.57 ± 0.10 nM/mg of protein. Untreated animals (Group II) showed a marked increase (*p* < 0.001) in TBARS levels in the brain when compared with the control group (Group I). After treatment with rivastigmine, there was a significant (*p* < 0.001) reduction in TBARS in the brain when compared to aluminum chloride-treated animals (Group II). D-(-)-Quinic acid-treated animals (Groups IV and V) had a marked decrease in TBARS (*p* < 0.001) when compared to Group II and the effect of D-(-)-quinic acid was dose dependent (Figure 7 and Table 4).

#### 3.2.6. Effect of D-(-)-Quinic Acid on Nitrite Levels in the Rat Brain

**Figure 8:**
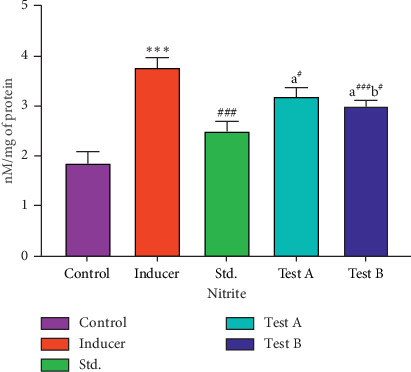
Effect of D-(-)-quinic acid on nitrite level in the rat brain. All values are represented as mean ± SEM.

Nitrite levels in the different groups are shown in Table 4. In the control group (Group I), the level of nitrite was found to be 1.87 ± 0.22 nM/mg. Aluminum chloride-treated rats showed a significant increase in nitrite levels in the brain (*p* < 0.001) when compared with the control group (Group I). The animals treated with rivastigmine (Group III) showed a significant (*p* < 0.001) reduction in nitrite within the brain when compared with the aluminum chloride-treated group (Group II). D-(-)-Quinic acid-treated animals (Groups IV and V) showed a significant decrease in the level of nitrite (*p* < 0.001) when compared to aluminum chloride-affected animals after 36 days (Figure 8 and Table 4).

#### 3.2.7. Effect of D-(-)-Quinic Acid on MAO Levels in the Rat Brain

The effect of D-(-)-quinic acid on MAO is shown in Table 5. The MAO-A and MAO-B levels in the control group were found to be 29.5 ± 1.42 and 25.4 ± 1.40 nmol/kg, respectively. Rivastigmine did not show any effect on MAO activity. D-(-)-Quinic acid showed a dose-dependent inhibition of MAO activity.

## 4. Discussion

**Figure 9:**
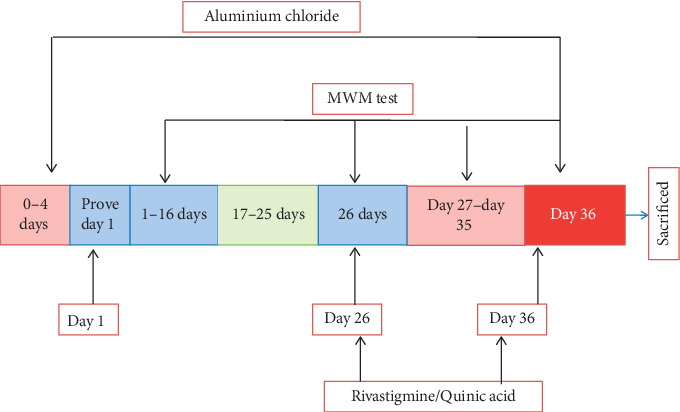
Systematic representation of experimentation protocol in aluminum chloride-induced dementia.

The results of the present study indicate that rats treated with aluminum chloride for 36 days exhibit cognitive deficits, abnormal biochemistry, and histological changes in the brain similar to that seen in dementia associated with AD [21]. Treatment with D-(-)-quinic acid was protective for cognition, biochemistry, and histology (Figure 9). Aluminum is a potent neurotoxin involved in the progression of various cognitive disorders. Chronic aluminum exposure induces oxidative stress, which may be a mechanism involved in AD [22, 23]. Aluminum chloride prolongs impairment of cognition and oxidative stress. In the brain, aluminum accumulates in various areas, such as the hippocampus and frontal cortex. It can lead to elevated amyloid precursor protein (APP) expression [24, 25] amyloid *β* (A*β*) deposition [26], impaired cholinergic transmission, apoptotic neuronal death, and increased phosphorylation of tau. Moreover, aluminum chloride leads to oxidative stress and deterioration of cellular lipid proteins and DNA.

In our study, cognition was assessed using the Morris water maze. Aluminum exposure resulted in a reduction in spatial memory. Furthermore, there was a significant increase in acetylcholinesterase (AChE) activity and oxidative stress in the brain, as indicated by the increased level of TBARS and the depletion of GSH [27].

In the present study, the administration of rivastigmine significantly improved memory deficit produced by aluminum chloride. Rivastigmine reduced AChE levels in the brain and attenuated oxidative damage alteration at a normal dose. Histopathologically, there was less neuronal damage following rivastigmine. The possible use of monoamine oxidase (MAO) inhibitors in AD was first reported by Charles. M in 1977. This study reported that MAO is involved in the neuronal damage and production of H_2_O_2_, which is a possible source of oxidative stress. Several other studies have described an increased activity of MAO-B in the brain and platelets of patients suffering from neurodegenerative disease, such as AD or Parkinson's disease [28, 29]. Our study also observed increased levels of MAO-B in the rat brain.

In our study, we saw neutrophil infiltration and blood vessel congestion into the brain as seen through histology following aluminum chloride treatment. Vascular necrosis may indicate an accelerated neurodegeneration and increased inflammatory events following AlCl_3_ [30].

In the present study, administration of D-(-)-quinic acid reduced memory deficits caused by AlCl_3_ administration. In addition, we also reported D-(-)-quinic acid was associated with anti-AChE activity and attenuated oxidative alteration and brain MAO levels which occurred in a dose-dependent manner. D-(-)-Quinic acid-treated animals exhibited reduced histopathological alterations, which is an indication of the protective effects of D-(-)-quinic acid against aluminum chloride-induced dementia of the AD type.

## 5. Conclusions

The present findings suggest that quinic acid exhibits neuroprotective effects for aluminum chloride-induced dementia. The behavioral impairments caused by aluminum chloride were significantly attenuated by quinic acid. Histopathological studies of the hippocampus and cortex also indicated that quinic acid markedly reduced the toxicity of AlCl_3_ and preserved the normal histoarchitecture of the hippocampus and cortex. Moreover, quinic acid reversed memory deficits caused by aluminum chloride through decreased in AChE activity and TBARS and increased antioxidant enzyme activity (GSH, catalase, and SOD). Our results suggest that quinic acid might be beneficial in the prevention of dementia development and progression.

## Figures and Tables

**Table 1:** Effect of D-(-)-quinic acid on aluminum chloride-induced dementia in rats using the Morris water maze.

Groups/treatments	ELT (sec)			
	5th day	16th day	26th day	36th day
I (control): 0.9% NaCl (5 ml/kg, p.o.)	3.77 ± 0.09	4.27 ± 0.21	4.75 ± 0.17	5.32 ± 0.42
II (inducer): AlCl_3_ (175 mg/kg, p.o.)	3.76 ± 0.11	9.66 ± 0.34	13.61 ± 0.23	17.85 ± 2.14^*∗∗∗*^
II (standard): AlCl_3_ (175 mg/kg, p.o.) + rivastigmine (2.5 mg/kg)	3.69 ± 0.32	8.98 ± 0.52	11.44 ± 0.24	6.82 ± 0.67^###^
IV (test A): AlCl_3_ (175 mg/kg, p.o.) + D-(-)-quinic acid (200 mg/kg, p.o.)	3.81 ± 0.57	9.15 ± 0.26	10.27 ± 0.11	9.25 ± 0.54^a^^##^
V (test B): AlCl_3_ (175 mg/kg, p.o.) + D-(-)-quinic acid (400 mg/kg, p.o.)	3.77 ± 0.37	9.34 ± 0.17	11.04 ± 0.32	8.72 ± 0.67^a^^###^

Data are presented as mean ± SEM values, *n* = 6. Data were analyzed by one-way ANOVA followed by the Tukey -Kramer multiple comparison test and the significance of results showed as ^∗^*p* < 0.05, ^∗∗^*p* < 0.01, ^∗∗∗^*p* < 0.001, ^ns^*p* > 0.05 when compared with the normal control. ^#^*p* < 0.05, ^##^*p* < 0.01, ^###^*p* < 0.001 when compared with the normal control, 1, ^a^when compared with the inducer, and ^b^when compared with the standard.

**Table 2:** Effect of D-(-)-quinic acid on aluminum chloride-induced dementia in rats using the Morris water maze.

Groups/treatments	TT (sec)			
	5th day	16th day	26th day	36th day
I (control): 0.9% NaCl (5 ml/kg, p.o.)	26.18 ± 0.11	28.19 ± 0.10	27.24 ± 0.092]	30.34 ± 0.35
II (inducer): AlCl_3_ (175 mg/kg, p.o.)	24.11 ± 0.08	17.95 ± 0.08	15.42 ± 0.18	12.34 ± 0.36^*∗∗∗*^
III (standard): AlCl_3_ (175 mg/kg, p.o.) + rivastigmine (2.5 mg/kg, p.o.)	22.62 ± 0.09	19.25 ± 0.17	21.75 ± 0.25	23.97 ± 0.16^a^^###^
IV (test A): AlCl_3_ (175 mg/kg, p.o.) + D-(-)-quinic acid (200 mg/kg, p.o.)	23.18 ± 0.13	18.81 ± 0.07	18.43 ± 0.17	21.76 ± 0.25^a^^###^
V (test B): AlCl_3_ (175 mg/kg, p.o.) + D-(-)-quinic acid (400 mg/kg, p.o.)	24.70 ± 0.25	20.54 ± 0.13	21.70 ± 0.09	26.75 ± 0.29^a^^###^^b ns^

Data are presented as mean ± SEM values, n = 6. Data were analyzed by one-way ANOVA followed by the Tukey -Kramer multiple comparison test and the significance of results showed as ^∗^*p* < 0.05, ^∗∗^*p* < 0.01, ^∗∗∗^*p* < 0.001, ^ns^*p* > 0.05 when compared with the normal control. ^#^*p* < 0.05, ^##^*p* < 0.01 , ^###^*p* < 0.001 when compared with the normal control, 1, ^a^when compared with the inducer, and ^b^when compared with the standard.

**Table 3:** Effect of D-(-)-quinic acid on aluminum chloride-induced dementia in rats as indicated using an actophotometer.

Groups/treatments	LA (count/10 min) on 5th day	LA (count/10 min) on 16th day	LA (count/10 min) on 26th day	LA (count/10 min) on 36th day
I (control): 0.9% NaCl (5 ml/kg, p.o.)	150.11 ± 1.24	159.34 ± 0.93	155.43 ± 0.91	151.22 ± 1.68
II (inducer): AlCl_3_ (175 mg/kg, p.o.)	163.47 ± 2.37	89.58 ± 0.63	82.10 ± 1.01	76.82 ± 1.85^*∗∗∗*^
III (std.): AlCl_3_ (175 mg/kg, p.o.)+ rivastigmine (2.5 mg/kg)	145.72 ± 1.03	86.17 ± 0.36	83.09 ± 0.89	143.09 ± 2.46^###^
IV (test A): AlCl_3_ (175 mg/kg, p.o.) + D-(-)-quinic acid (200 mg/kg, p.o.)	147.29 ± 0.82	84.90 ± 0.45	82.59 ± 0.79	100.49 ± 1.93^a^^##^^b^^#^
V (test B): AlCl_3_ (175 mg/kg, p.o.) + D-(-)-quinic acid (400 mg/kg, p.o.)	159.98 ± 1.46	83.75 ± 0.72	83.30 ± 0.67	123.39 ± 2.14^a^^###^^b^^##^

Data are presented as mean ± SEM values, *n* = 6. Data were analyzed by one-way ANOVA followed by the Tukey–Kramer multiple comparison test ^*∗*^*p* < 0.05, ^*∗∗*^*p* < 0.01, ^*∗∗∗*^*p* < 0.001, ^ns^*p* > 0.05 when compared with the normal control, ^#^*p* < 0.05, ^##^*p* < 0.01, ^###^*p* < 0.001, ^a^when compared with the inducer, and ^b^when compared with the standard.

**Table 4:** Effect of D-(-)-quinic acid on aluminum chloride-induced dementia in rats as indicated by biochemical parameters.

Groups/treatments	AChE (nM/L/gm of tissue)	GSH (nM/mg of protein)	Catalase (*µ*M of H_2_O_2_ decomposed/min/mg of protein)	SOD (units/mg of protein)	TBARS (nM/mg of protein)	Nitrite (nM/mg of protein)
I (control): 0.9% NaCl (5 ml/kg, p.o.)	1.39 ± 0.09	2.50 ± 0.10	1.87 ± 0.15	4.77 ± 0.20	0.57 ± 0.10	1.87 ± 0.22
II (inducer): AlCl_3_ (175 mg/kg, p.o.)	3.50 ± 0.15^*∗∗∗*^	0.90 ± 0.04^*∗∗∗*^	0.64 ± 0.16^*∗∗∗*^	1.00 ± 0.10^*∗∗∗*^	1.38 ± 0.05^*∗∗∗*^	3.79 ± 0.20^*∗∗∗*^
III (standard): AlCl_3_ (175 mg/kg, p.o.) + rivastigmine (2.5 mg/kg, p.o.)	1.78 ± 0.15^###^	2.09 ± 0.05^###^	1.61 ± 0.19^###^	4.20 ± 0.22^###^	0.61 ± 0.05^###^	2.52 ± 0.15^###^
IV (test A): AlCl_3_ (175 mg/kg, p.o.) + D-(-)-quinic acid (200 mg/kg, p.o.)	2.73 ± 0.07^a^^##^	1.87 ± 0.15^a^^##^	0.81 ± 0.22a ns b#	3.21 ± 0.17^a^^##^	1.19 ± 0.03^a^^###^	3.21 ± 0.17^a^^#^
V (test B): AlCl_3_ (175 mg/kg, p.o.) + D-(-)-quinic acid (400 mg/kg, p.o.)	2.22 ± 0.15^a^^###^ b#	1.96 ± 0.15^a^^###^^b^^##^	1.024 ± 0.13^a^^###^^b^ ns	3.99 ± 0.12^a^^###^	0.88 ± 0.04^a^^###^	3.03 ± 0.10^a^^###^^b^^#^

Data are presented as mean ± SEM values, *n* = 6. Data were analyzed by one-way ANOVA followed by the Tukey–Kramer multiple comparison test ^*∗*^*p* < 0.05, ^*∗∗*^*p* < 0.01, ^*∗∗∗*^*p* < 0.001, ^ns^*p* > 0.05 when compared with the normal control, ^#^*p* < 0.05, ^##^*p* < 0.01, ^###^*p* < 0.001, ^a^when compared with the inducer, and ^b^when compared with the standard.

**Table 5:** Effect of D-(-)-quinic acid on monoamine oxidase (MAO) level in the rat brain.

Groups/treatments	MAO-A (nmol/mg of protein)	MAO-A inhibition (%)	MAO-B (nmol/mg of protein)	MAO-B
Inhibition (%)				
I (control): 0.9% NaCl (5 ml/kg, p.o.)	29.5 ± 1.42		25.4 ± 1.48	
II (inducer): AlCl_3_ (175 mg/kg, p.o.)	39.8 ± 1.28	0.00	34.5 ± 0.61	0.00
III (std.): AlCl_3_ (175 mg/kg, p.o.) + rivastigmine (2.5 mg/kg)	34.5 ± 0.92 ^ns^	14	30.6 ± 1.81 ^ns^	12.4
IV (test A): AlCl_3_ (175 mg/kg, p.o.) + D-(-)-quinic acid (200 mg/kg, p.o.)	32.5 ± 1.12^*∗*^	18.4	28.3 ± 0.51^*∗*^	17.8
V (test B): AlCl_3_ (175 mg/kg, p.o.) + D-(-)-quinic acid (400 mg/kg, p.o.)	30.1 ± 1.41^*∗∗*^	23.7	26.5 ± 1.41^*∗∗*^	23.2

Data are presented as mean ± SEM values, *n* = 6. Data were analyzed by one-way ANOVA followed by the Tukey -Kramer multiple comparison test ^∗^*p* < 0.05, ^∗∗^*p* < 0.01, ^∗∗∗^*p* < 0.001, ns *p* > 0.05 when compared with the normal control.

## Data Availability

The data can be made available on journal request.

## References

[1] Gatz M., Mortimer J. A., Fratiglioni L., Johansson B., Berg S., Andel R. (2007). Accounting for the relationship between low education and dementia: a twin study. *Physiology and Behavioyr*.

[2] Reitz C., Brayne C., Mayeux R. (2011). Epidemiology of alzheimer disease. *Nature Reviews Neurology*.

[3] Ferri C. P., Prince M., Brayne C. (2005). Global prevalence of dementia: a Delphi consensus study. *The Lancet*.

[4] Overshott R., Burns A. (2007). Clinical assessment in dementia. *Psychiatry*.

[5] Chapman D. P., Williams S. M., Strine T. W., Anda R. F., Moore M. J. (2006). Dementia and its implications for public health. *Preventing Chronic Disease*.

[6] Herrmann K. M. (1995). The shikimate pathway: early steps in the biosynthesis of aromatic compounds. *The Plant Cell*.

[7] Pero R. W., Harald L., Tomas L. (2008). Antioxidant metabolism induced by quinic acid increased urinary excretion of tryptophan and nicotinamide. *Phytotherapy Research*.

[8] Devi B., Bais S., Gill N. S. (2017). A Review on quinic acid and its therapeutic potential. *Inventi Rapid: Molecular Pharmacology*.

[9] Mcgheer P., Mcgheer E. G. (2001). Inflammation cytoxicity and Alzheimer’s disease. *Neurology Aging*.

[10] Schuchang H. (2008). Protective effects of gastrodia elata on aluminium chloride-induced learning impairments and alterations of amino acid neurotransmitter release in adult rats. *Restorative Neurology and Neuroscience*.

[11] Ellman G. L., Courtney K. D., Andres V., Featherstone R. M. (1961). A new and rapid colorimetric determination of acetylcholinesterase activity. *Biochemical Pharmacology*.

[12] Morris R. (1984). Developments of a water-maze procedure for studying spatial learning in the rat. *Journal of Neuroscience Methods*.

[13] Beutler E., Duron O., Kelley B. M. (1963). Improved methods for determination of blood glutathione. *The Journal of Laboratory and Clinical Medicine*.

[14] Jollow D. J., Mitchell J. R., Zampaglione N., Gillette J. R. (1974). Bromobenzene-induced liver necrosis. protective role of glutathione and evidence for 3,4-bromobenzene oxide as the hepatotoxic metabolite. *Pharmacology*.

[15] Luck H., Bergmeyer H. U. (1971). *Catalase: Methods of Enzymatic Analysis*.

[16] Armstrong D. M., Ikonomovic M. D., Sheffield R., Wenthold R. J. (1994). AMPA-selective glutamate receptor subtype immunoreactivity in the entorhinal cortex of non-demented elderly and patients with Alzheimer’s disease. *Brain Research*.

[17] Ohkawa H., Ohishi N., Yagi K. (1979). Assay for lipid peroxides in animal tissues by thiobarbituric acid reaction. *Analytical Biochemistry*.

[18] Yagi K. (1998). Simple procedure for specific assay of lipid hydroperoxides in serum of plasma. *Free Radical and Antioxidant Protocols*.

[19] Lyle N., Dipankar B., Tapas K. S. (2009). Stress modulating antioxidant effect of *Nardostachys jatamansi*. *Indian Journal of Biochemistry and Biophysics*.

[20] Kangtao Y., Bais S. (2018). Neuroprotective effect of protocatechuic acid through MAO-B inhibition in aluminium chloride induced dementia of Alzheimer’s type in rats. *International Journal of Pharmacology*.

[21] Charles M., McEwen J., Tabor H., Tabor C. W. (1977). Mao activity in rabbit serum. *Methods in Enzymologists*.

[22] Lin R., Chen X., Li W., Han Y., Liu P., Pi R. (2008). Exposure to metal ions regulates mRNA levels of APP and BACE1 in PC12 cells: blockage by curcumin. *Neuroscience Letters*.

[23] Walton J. R., Wang M.-X. (2009). APP expression, distribution and accumulation are altered by aluminum in a rodent model for Alzheimer’s disease. *Journal of Inorganic Biochemistry*.

[24] Campbell A. (2002). The potential role of aluminium in Alzheimer’s disease. *Nephrology Dialysis Transplantation*.

[25] Abdel-Aal R. A., Assi A. A. A., Kostandy B. B. (2011). Rivastigmine reverses aluminum-induced behavioral changes in rats. *European Journal of Pharmacology*.

[26] Stevenson L., Phillips F., O’sullivan K., Walton J. (2012). Wheat bran: its composition and benefits to health, a European perspective. *International Journal of Food Sciences and Nutrition*.

[27] Bharathi P., Shamasundar N. M., Sathyanarayana Rao T. S., Dhanunjaya Naidu M., Ravid R., Rao K. S. J. (2006). A new insight on Al-maltolate-treated aged rabbit as Alzheimer’s animal model. *Brain Research Reviews*.

[28] Adolfsson R., Gottfries C.-G., Oreland L., Wiberg Å., Winblad B. (1980). Increased activity of brain and platelet monoamine oxidase in dementia of Alzheimer type. *Life Sciences*.

[29] Bais S., Kumari R. (2018). Modulatory effect of sinapic acid in toluene induced dementia of leukoencephalopathy type in wistar rats: a biochemical study. *The Natural Products Journal*.

[30] Bais S., Kumari R., Prashar Y. (2018). Ameliorative effect of trans-sinapic acid and its protective role in cerebral hypoxia in aluminium chloride induced dementia of alzheimer’s type. *CNS and Neurological Disorders - Drug Targets*.

